# A mutant of phosphomannomutase1 retains full enzymatic activity, but is not activated by IMP: Possible implications for the disease PMM2-CDG

**DOI:** 10.1371/journal.pone.0189629

**Published:** 2017-12-19

**Authors:** Valentina Citro, Chiara Cimmaruta, Ludovica Liguori, Gaetano Viscido, Maria Vittoria Cubellis, Giuseppina Andreotti

**Affiliations:** 1 Dipartimento di Biologia, Università Federico II, Napoli, Italy; 2 Istituto di Chimica Biomolecolare–CNR, Pozzuoli, Italy; 3 Dipartimento di scienze e tecnologie ambientali, biologiche e farmaceutiche, Università della Campania "Luigi Vanvitelli", Caserta, Italy; George Washington University, UNITED STATES

## Abstract

The most frequent disorder of glycosylation, PMM2-CDG, is caused by a deficiency of phosphomannomutase activity. In humans two paralogous enzymes exist, both of them require mannose 1,6-bis-phosphate or glucose 1,6-bis-phosphate as activators, but only phospho-mannomutase1 hydrolyzes bis-phosphate hexoses. Mutations in the gene encoding phosphomannomutase2 are responsible for PMM2-CDG. Although not directly causative of the disease, the role of the paralogous enzyme in the disease should be clarified. Phosphomannomutase1 could have a beneficial effect, contributing to mannose 6-phosphate isomerization, or a detrimental effect, hydrolyzing the bis-phosphate hexose activator. A pivotal role in regulating mannose-1phosphate production and ultimately protein glycosylation might be played by inosine monophosphate that enhances the phosphatase activity of phosphomannomutase1. In this paper we analyzed human phosphomannomutases by conventional enzymatic assays as well as by novel techniques such as ^31^P-NMR and thermal shift assay. We characterized a triple mutant of phospomannomutase1 that retains mutase and phosphatase activity, but is unable to bind inosine monophosphate.

## Introduction

PMM2-CDG (MIM#212065), also known as CDG-1A or Jaeken syndrome, is an autosomic recessive disease without a cure [1]. The recommended name of this pathology underlines the fact that it is caused by mutations in the gene *PMM2* encoding phosphomannomutase2 (UniProt: PMM2_HUMAN)[2]. The first step towards protein N- or C-glycosylation requires the conversion of mannose 6-phosphate (Man-6-P) into mannose 1-phosphate (Man-1-P). Man-1-P is a precursor to GDP-mannose necessary for the synthesis of dolichol phosphate mannose and lipid-linked oligosaccharides [3, 4]. In humans there are two paralogous enzymes, PMM1 and PMM2 [5]. Both enzymes require an activator, glucose 1,6 bisphosphate (Glc-1,6-P_2_) or mannose 1,6 bisphosphate (Man-1,6-P_2_) and catalyse the conversion of glucose 1-phosphate (Glc-1-P) into glucose 6-phosphate (Glc-6-P) beside that of Man-1-P into Man-6-P [6]. Only PMM1 has an additional phosphatase activity and is able to hydrolyze Glc-1,6-P_2_ and Man-1,6-P_2_. This latter activity is enhanced by increased concentrations of Inosine monophosphate (IMP) [7]. Mutations of *PMM2* cause PMM2-CDG. Patients are either homozygous for a hypomorphic mutation or are compound heterozygous with one inactivating and one hypomorphic mutation. The residual activity of several mutants has been measured [8]. Homozygous inactivating mutations of *PMM2* have never been observed in humans possibly because total absence of this enzyme is not compatible with life[9]. On the contrary, mutations of *PMM1* have never been associated to human diseases. These findings are confirmed in mice where disruption PMM2 causes early embryonic lethality [10] whereas that of PMM1, which is widely expressed in embryos, has no apparent deleterious effects[11]. PMM1 is down-regulated in adults, but it remains highly expressed in brain and is detectable in liver, lungs, pancreas and endocrinal glands [11]. Unfortunately the effect of PMM1 mutations on hypomorphic PMM2 has not been evaluated yet. In yeast, *PMM1* restores growth at the restrictive temperature in cells harbouring the conditional lethal sec53-6 allele [12]. *PMM1* and *PMM2* evolved by gene duplication and are located on chromosomes 16p13 [13] and 22q11-13 respectively [14, 15] while a processed pseudo-gene similar to *PMM2* is present on chromosome 18 [14]. Their protein products share 63.7% identical amino-acids. A subset of amino-acids are highly conserved or invariant within PMM1 and PMM2 clusters, but not between the clusters, might account for the functional differences observed between the paralogous enzymes [15]. The x-ray structure of PMM1 was solved in absence of sugar ligands (2FUC) and in presence of Man-1-P (2FUE) [16], that of PMM2 (2AMY) was solved in the absence of ligands, but a model of the protein complexed with Glc-1,6-P_2_ was recently obtained [17].

With this paper we wish to contribute to a better understanding of the specific roles of the two enzymes. Using ^31^P-NMR we could demonstrate that PMM1 as well as PMM2 convert Man-6-P into Man-1-P, this activity had never been monitored directly before. In fact the assays which have been used so far, study the reaction in the non-biological relevant direction (i.e. Man-1-P to Man-6-P), dosing the oxidation of Glu-6-P which is obtained from Man-6-P thanks to ancillary enzymes [6, 18–21]. Using ^31^P-NMR we measured the equilibrium constant of mannose-phosphates interconversion too. We demonstrated that IMP is a competitive inhibitor of the mutase activity of PMM1, but activates its phosphatase activity. This is peculiar, since we identified another molecule, the FDA approved drug clodronate, that inhibits both activities. Clodronate recognizes specifically PMM1 and has no effect on PMM2. Although only an X-ray structure of the PMM1-IMP complex could permit to elucidate the interactions between the protein and the nucleotide completely, by modelling and site directed mutagenesis we identified the residues that are primarily responsible for IMP binding. We showed that the triple mutant M186Q-N218D-E219K-PMM1 (QDK-PMM1) is a stable protein that retains mutase and phosphatase, but is not sensitive to IMP. The binding of IMP to PMM1 was modelled with PELE [22, 23], a novel computational tool to predict flexible ligand protein interactions.

## Methods and materials

DEAE-Sepharose ff, Superdex-75, Butyl Sepharose ff, were purchased from GE Healthcare Life Sciences. Phosphoglucose isomerase from rabbit muscle, phosphomannose isomerase from *E*. *coli*, glucose 6-phosphate dehydrogenase from baker's yeast (*S*. *cerevisiae*), phosphoglucomutase from rabbit muscle, α-D-Glucose 1,6-bisphosphate potassium salt hydrate, α-D-Glucose 1-phosphate disodium salt hydrate, α-D(+)Mannose 1-phosphate sodium salt hydrate, D-Glucose 6-phosphate, D-Mannose 6-phosphate, β-Nicotinamide adenine dinucleotide phosphate sodium salt, were purchased from Sigma-Aldrich. Sypro Orange was from Invitrogen Molecular Probes. Sodium orthovanadate was from Acros Organics. Creatine phosphate disodium salt tetrahydrate was from Alfa Aesar. Clodronate ((Dichloro-phosphono-methyl)phosphonic acid) and neridronate ((6-Amino-1-hydroxyhexane-1,1-diyl)bis(phosphonic acid) were from ABIOGEN-PHARMA.

Mannose 1,6-bisphosphate was synthesized as described [18] and purified on an AG1x8 hydroxide form column by running a step gradient 0–1 M NaCl. Fractions were analyzed by ^1^H- and ^31^P-NMR spectroscopy. An internal standard (Trimethylsilylpropanoic acid) was used in order to measure the concentration. All the other reagents were of analytical grade. The ORF encoding QDK-PMM1 was obtained by *de novo* gene synthesis and was purchased by GeneCust, Luxemburg; it is available upon request.

### Protein expression and purification

Wild type PMM2 and PMM1, and QDK-PMM1 were expressed in *E*. *coli* BL21(DE3) strain grown at 37°C in LB broth containing ampicillin 0.2 mg/ml. The expression and purification of wild type was performed as described [16, 24, 25], with only minor changes. The expression of QDK-PMM1 was assessed. The best production of the protein was obtained by adding IPTG 0.4 mM when the optical density was 0.1 and prolonging the incubation for four hours after induction. The cells were then harvested, washed with PBS, enzymatically lysed with lysozyme 1 mg/ml (in Hepes 50 mM pH 7.5 containing 0.1 mM 2-mercaptoethanol, 1 mM EDTA, 0.1 mM phenylmethylsulfonyl fluoride, 5% glycerol), treated with Deoxyribonuclease I 0.005 mg/ml, and ammonium sulphate was added to the clear homogenate up to 50% saturation. The precipitate was recovered, dissolved in buffer, dialyzed against Hepes 50 mM pH 7.1 containing 5 mM MgCl_2_, 1 mM 2-mercaptoethanol, 1% glycerol, and loaded onto a DEAE-Sepharose ff column equilibrated with the same buffer. A gradient (0–0.7 M NaCl) was applied. Functional assay has been carried out to isolate specific fractions that were dialyzed. The sample, after the addition of 20% ammonium sulphate, was loaded onto a Butyl-Sepharose ff column equilibrated with Tris 50 mM pH 7.1 containing 5 mM MgCl_2_, 1 mM 2-mercaptoethanol, 20% ammonium sulphate, 5% glycerol. A gradient (20–0% ammonium sulphate) was applied and the active fractions, judged pure by SDS-PAGE, were pooled, dialyzed (in buffer containing 5% glycerol), concentrated and stored at -20°C.

### Enzyme assay

PMM1 and PMM2 have three enzymatic activities:

$$hexose\text{-}1,6\text{-}P_{2}+PMM=hexose\text{-}6P\;\left(or\;hexose\text{-}1P\right)+PMM\text{-}P$$ (1)

$$hexose\text{-}1P\;\left(or\;hexose\text{-}6P\right)+PMM\text{-}P=hexose\text{-}1,6\text{-}P_{2}+PMM$$ (2)

$$PMM\text{-}P+H_{2}O=PMM+P_{i}$$ (3)

Phosphomannomutaseactivity:(1)+(2),hexose=mannose

Phosphoglucomutaseactivity:(1)+(2),hexose=glucose

Phosphataseactivity:(1)+(3),hexose=mannoseorglucose

All the enzymatic activities were routinely measured at 32°C in Hepes 20 mM pH 7.5 containing MgCl_2_ 5 mM, NaCl 150 mM, NADP^+^ 0.25 mM, BSA 0.1 mg/ml in the presence of the appropriate concentrations of substrates/activators (specified time by time) and the required ancillary enzymes.

In particular, phosphomannomutase activity (by using Man-1-P as the substrate and Man-1,6-P_2_ or Glc-1,6-P_2_ as the activator) was measured in the presence of 2.6 U/ml glucose 6-phosphate dehydrogenase, 3.7 U/ml phosphoglucose isomerase and 0.003 mg/ml phosphomannose isomerase.

Phosphoglucomutase activity (by using Glc-1-P as the substrate and Glc-1,6-P_2_ as the activator) was measured in the presence of 2.6 U/ml glucose 6-phosphate dehydrogenase.

Phosphatase activity was measured in the presence of Man-1,6-P_2_ adding 2.6 U/ml glucose 6-phosphate dehydrogenase, 3.7 U/ml phosphoglucose isomerase and 0.003 mg/ml phosphomannose isomerase. Alternatively phosphatase activity was measured in the presence of Glc-1,6-P_2_ adding 2.6 U/ml glucose 6-phosphate dehydrogenase and 0.034 U/ml phosphoglucomutase from rabbit muscle. In both cases, the activity was also measured in the presence of 0.17 mM of IMP.

In any case, the activity was followed spectrophotometrically at 340 nm, recording the reduction of NADP^+^ to NADPH.

### ^31^P NMR spectroscopy

The ^1^H-decoupled, one-dimensional ^31^P spectra were recorded at 161.976 MHz on a Bruker AVANCE™III HD spectrometer 400MHz, equipped with a BBO BB-H&F-D CryoProbeTM Prodigy fitted with a gradient along the Z-axis, at a probe temperature of 27°C. This temperature, which is lower than that used in spectrophotometric assays, was chosen to minimize the inactivation of the enzymes during the accumulation of spectra. Spectral width 120 ppm, delay time 1.2 sec, pulse width of 12.0 μs were applied.

Samples contained 10% ^2^H2O for internal lock, and phosphocreatine was added as an internal standard (0 ppm).

### Thermal stability

Melting profiles were recorded under different conditions by thermal shift assay with the StepOne Real-Time PCR System (Applied Biosystems)[26]. The proteins (PMM2, PMM1 or QDK-PMM1, 0.5 mg/mL final concentration) were equilibrated in Hepes 20 mM pH 7.5, MgCl_2_ 1 mM, NaCl 150 mM, dithiothreitol 1 mM, Sypro Orange 2.4x, then distributed in 0.2 ml PCR-strip (compatible with the instrument). The appropriate ligand solution (in water) was added (the final volume was 0.025 ml each), and then the strips were sealed and heated from 20 to 90° at 1°C/min with increments of 0.6°C.

The experiment was performed on PMM1 and PMM2 in the presence of Glc-1,6-P_2_ ranging from 0 to 1 mM. PMM1 and QDK-PMM1were analyzed in the presence of: no ligand, Glc-1-P 0.5 mM, Glc-1-P 0.5 mM plus vanadate 0.5 mM, vanadate 0.5 mM, Glc-1,6-P_2_ 0.5 mM, IMP 0.17 mM.

### Docking

The starting structure for docking was 2FUE[16]. The X-ray structure deposited in the PDB contains Man-1-P and a single chain in the asymmetric unit. The ligand was removed and the dimeric biological assembly was obtained with the PISA server [27]. The hydrogen bond network of the initial model was optimized with the Protein Wizard optimization from Maestro at pH 7, [28]. Five different initial ligand positions were prepared by placing the ligand randomly in the solvent. Ligand binding was simulated with PELE[22, 23] essentially as described [17]. A global unconstrained search was followed by a local refinement search.

The unconstrained ligand search was carried out under default conditions[29]. The search is performed by equally combining (50% chance) long, 6 Å, and short, 0.75 Å, ligand perturbation (translation) steps. Rotations were kept in the [0:90] range. Furthermore, a randomly chosen ligand perturbation direction is kept for two Monte Carlo steps, allowing a more complete exploration of the entire protein surface. ANM perturbation includes the lowest 6 modes, with maximum displacements of the alpha carbon of 0.75 Å. A randomly chosen mode is kept for 6 steps to facilitate the coupling of ligand migration with large protein conformational changes. Side chain prediction is performed for all those residues having an atom within 5 Å of the ligand. The local search used translations of 0.4 Å and rotations in the [0:180] range. In addition, the randomly chosen ANM mode is only kept for 3 steps. For each accepted Monte Carlo step, the ligand’s binding energy is estimated by computing the protein-ligand interaction energy at the given geometry of the protein-ligand complex; PELE uses a OPLS-AA force field[30] with a surface area variable dielectric implicit solvent. Clearly, these energies do not aim to reproduce absolute binding free energies but to identify minima.

### Miscellaneous

The Bradford colorimetric assay was applied for protein quantification [31], using the Quick Start Bradford (Bio-Rad), with bovine serum albumin as standard.

Active site residues were identified by similarity with those of human PMM2, which in turn were identified on the model of the enzyme in closed conformation [17] with DrosteP [32]. The figures describing the interactions between PMM1 and IMP were prepared with CHIMERA [33] or LigPlot+[34]. At least 3 experiments were carried out to calculate standard deviations.

## Results

### Functional characterization by ^31^P NMR

The activity of PMM1 and PMM2 was analysed recording ^31^P-NMR spectra. These experiments require the accumulation of several scans for each NMR spectrum in order to have a good S/N ratio. They cannot be used to measure initial velocities, but have the advantage of monitoring all the species containing ^31^P.

The experiments were carried out recording the spectrum of the substrates, adding the enzyme to the substrates, sealing the NMR tube and starting the acquisition of the spectra.

**Fig 1:**
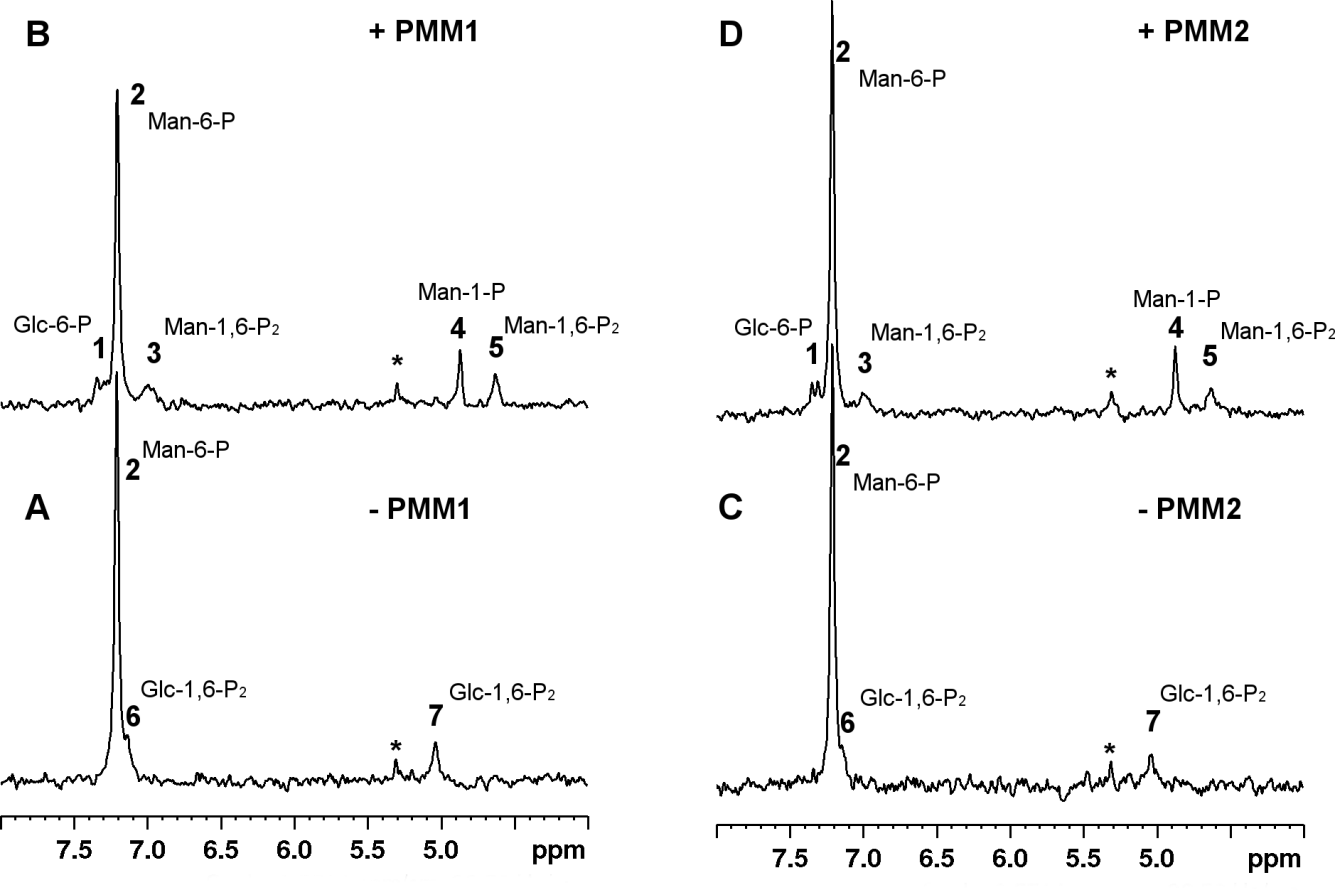
Phosphomannomutase activity of PMM1 and PMM2 on Man-6-P monitored by ^31^P-NMR spectroscopy. PMM1 or PMM2 (54 μg) were incubated with Man-6-P 1mM and Glc-1,6-P_2_ 0.1 mM at 27°C. Panel A) Spectrum of reagents prior to PMM1 addition. Panel B) Spectrum of products after PMM1 addition. Panel C) Spectrum of reagents prior to PMM2 addition. Panel D) Spectrum of products after PMM2 addition. The spectra were accumulated over 40 min. Creatine phosphate (1 mM) was added as an internal standard and all the spectra were referred to it (0 ppm). Resonance assignment was obtained by comparison with pure compounds: **1**, Glc-6-P; **2**, Man-6-P; **3** and **5**, Man-1,6-P_2_; **4**, Man-1-P; **6** and **7**, Glc-1,6-P_2_; *, inorganic phosphate.

Fig 1 shows the experiments carried out to monitor the isomerization Man-6-P 1 mM in the presence of Glc-1,6-P_2_ 0.1 mM: panels A and C are the spectra of substrates, panel B and D are the spectra accumulated for 40 min after the addition of PMM1 or PMM2 respectively.

When PMM1 (Fig 1, panel B) was added to the reaction mixture containing Man-6-P and Glc-1,6-P_2_ (Fig 1, panel A), the product Man-1-P (peak 4, Fig 1, panel B) as well as Man-1,6-P_2_ (peaks 3 and 5, Fig 1, panel B) are formed. The formation of Glc-6-P (peak 1, Fig 1 panels B and D) arising from Glc-1,6-P_2_ can also be observed.

A similar result was obtained with PMM2: when PMM2 (Fig 1, panel D) was added to the reaction mixture (Fig 1, panel C), the product Man-1-P (peak 4, Fig 1, panel D) as well as Man-1,6-P_2_ (peaks 3 and 5, Fig 1, panel D) are formed.

The signals of Man-1-P and Man-6-P were integrated and their ratio was calculated: the equilibrium is shifted towards the Man-6-P (Man-6-P/Man-1-P = 8.52±0.45). As expected, this value does not depend on the enzyme employed and it is stable over time. After 40 min the activator Man-1,6-P_2_, which is necessary for catalysis, is still present thus assuring that a constant concentration of the substrate and of the product is due to the attainment of the dynamic equilibrium and not to a halt of the enzyme. When the experiment is carried out with PMM1 and after a longer incubation, 330 min, the peaks of Man-1,6-P_2_ are not detectable.

**Fig 2:**
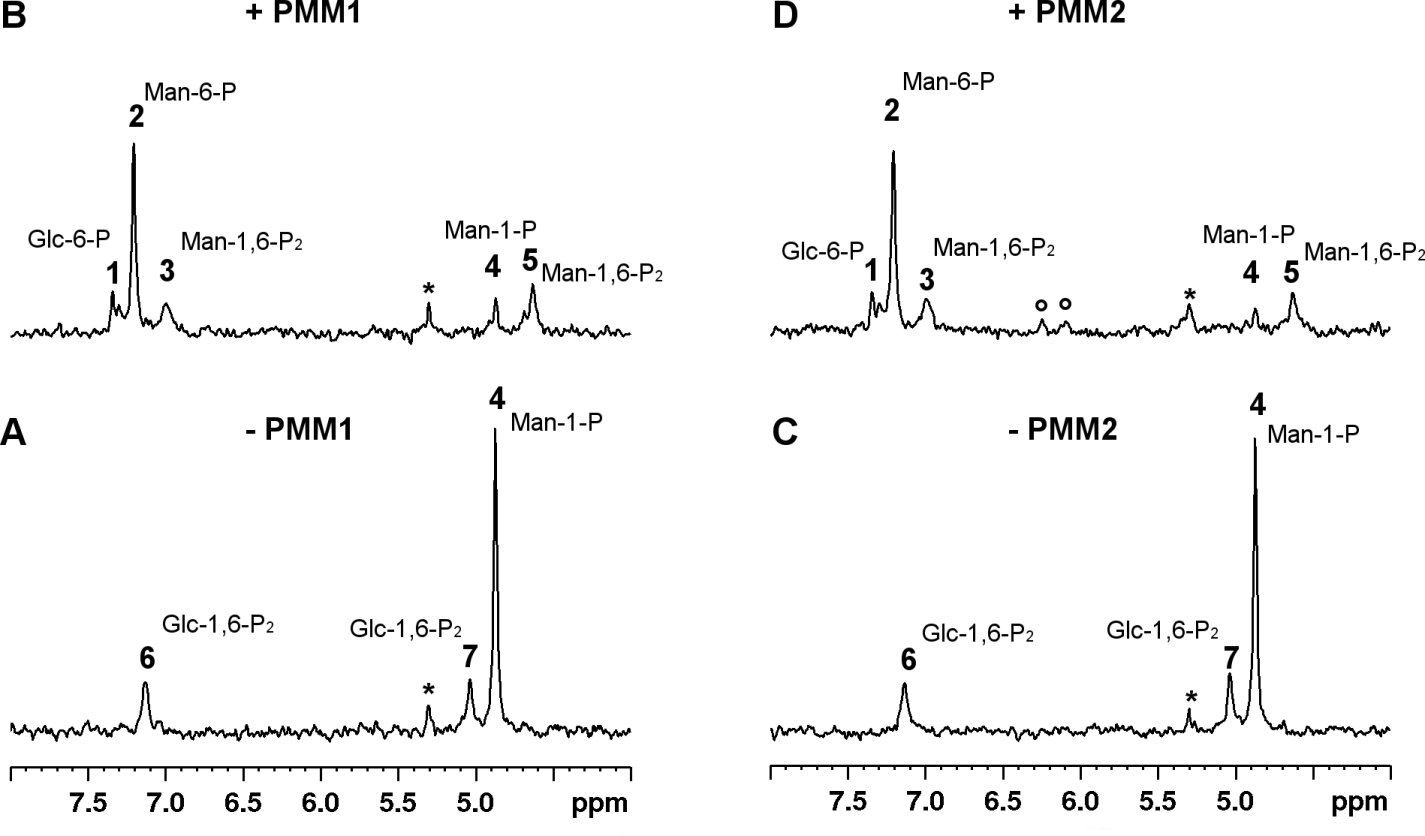
Phosphomannomutase activity of PMM1 and PMM2 on Man-1-P monitored by ^31^P-NMR spectroscopy. PMM1 or PMM2 (54 μg) were incubated with Man-1-P 0.5 mM and Glc-1,6-P_2_ 0.1 mM at 27°C, Panel A) Spectrum of reagents prior to PMM1 addition. Panel B) Spectrum of products after PMM1 addition. Panel C) Spectrum of reagents prior to PMM2 addition. Panel D) Spectrum of products after PMM2 addition. The spectra were accumulated over 40 min. Creatine phosphate (1 mM) was added as an internal standard and all the spectra were referred to it (0 ppm). Resonance assignment was obtained by comparison with pure compounds: **1**, Glc-6-P; **2**, Man-6-P; **3** and **5**, Man-1,6-P_2_; **4**, Man-1-P; **6** and **7**, Glc-1,6-P_2_; *, inorganic phosphate; °, unidentified peaks.

Fig 2 shows the experiments carried out to monitor the isomerization Man-1-P in the presence of Glc-1,6-P_2_: panels A and C are the spectra of substrates, panel B and D are the spectra accumulated for 40 minutes after the addition of PMM1 or PMM2 respectively.

When PMM1 was incubated with 0.5 mM Man-1-P and 0.1 mM Glc-1,6-P_2_, Man-6-P (peak 2, Fig 2, panel B) as well as Man-1,6-P_2_ (peaks 3 and 5, Fig 2, panel B) are formed.

The same happened with PMM2 (Fig 2, panel D): the formation of Man-6-P (peak 2) and Man-1,6-P_2_ (peaks 3 and 5) was recorded together with the accumulation of Glc-6-P (peak 1).

The equilibrium constant (Man-6-P/Man-1-P = 10.87±1.51) calculated in this direction (M6P->M1P) is comparable to that calculated in the opposite direction as expected.

The phosphatase activity of PMM1 was monitored by ^31^P NMR in the presence and in the absence of IMP. It is worth mentioning that the chemical shifts measured for inorganic phosphate (P_i_) and Glc-1-P are very similar at the pH used for enzymatic assays leading to the coalescence of the two peaks, hence the notation Glc-1-P+P_i_ will be used.

**Fig 3:**
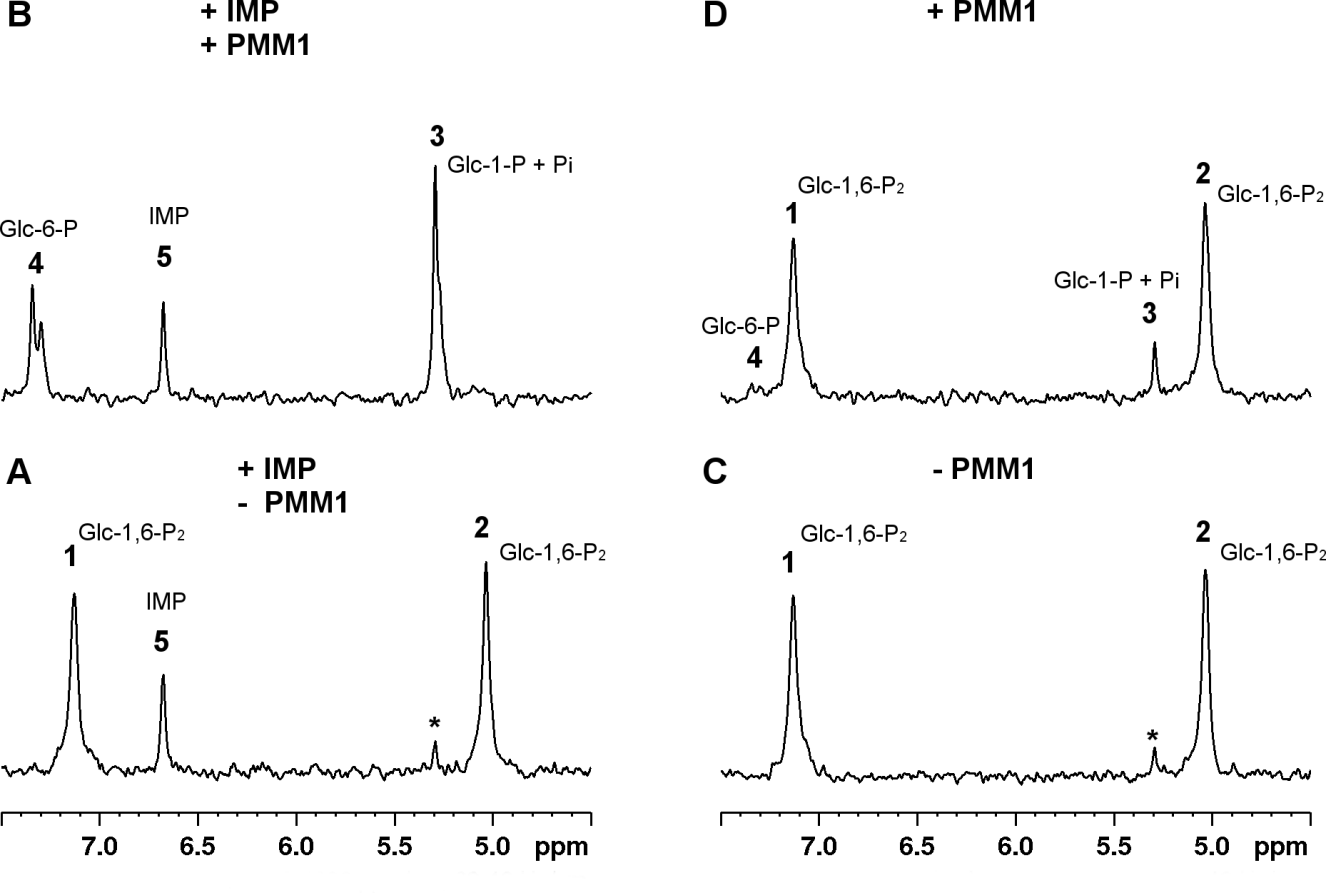
Phosphatase activity of PMM1 monitored by ^31^P-NMR spectroscopy. PMM1 (20 μg) was incubated with Glc-1,6-P_2_ 0.55 mM at 27°C, with and without IMP 0.17 mM. Panel A) Spectrum of reagents, Glc-1,6-P_2_ and IMP, prior to PMM1 addition. Panel B) Spectrum of products and IMP after PMM1 addition. Panel C) Spectrum of reagents, Glc-1,6-P_2_, prior to PMM1 addition. Panel D) Spectrum of products after PMM1 addition. Creatine phosphate (1 mM) was added as an internal standard and all the spectra were referred to it (0 ppm). Resonance assignment was obtained by comparison with pure compounds: **1** and **2**, Glc-1,6-P_2_; **3** Glc-1-P+P_i_; **4**, Glc-6-P; **5**, IMP; *, inorganic phosphate.

Fig 3 shows the experiments carried out to monitor the hydrolysis of Glc-1,6-P_2_ with or without IMP: panels A and C are the spectra of substrates, panel B and D are the spectra accumulated for 40 min after the addition of PMM1.

When PMM1 is incubated with Glc-1,6-P_2_ in the presence of IMP the complete consumption of Glc-1,6-P_2_ (peaks 1 and 2, Fig 3, panel A) accompanied by the formation of Glc-6-P (peak 4, Fig 3, panel B) and Glc-1-P+P_i_ (peak 3, Fig 3, panel B) is recorded. The experiment conducted in absence of IMP (Fig 3, panels C and D) showed the formation of only slight amounts of Glc-6-P (peak 4, panel D, Fig 3) and Glc-1-P+P_i_ (peak 3, Panel D, Fig 3), accompanied by a barely detectable consumption of Glc-1,6-P_2_. Under the same conditions, PMM2 did not hydrolyse Glc-1,6-P_2_ either in the presence or in the absence of IMP.

### Functional characterization by conventional enzymatic assays

**Fig 4:**
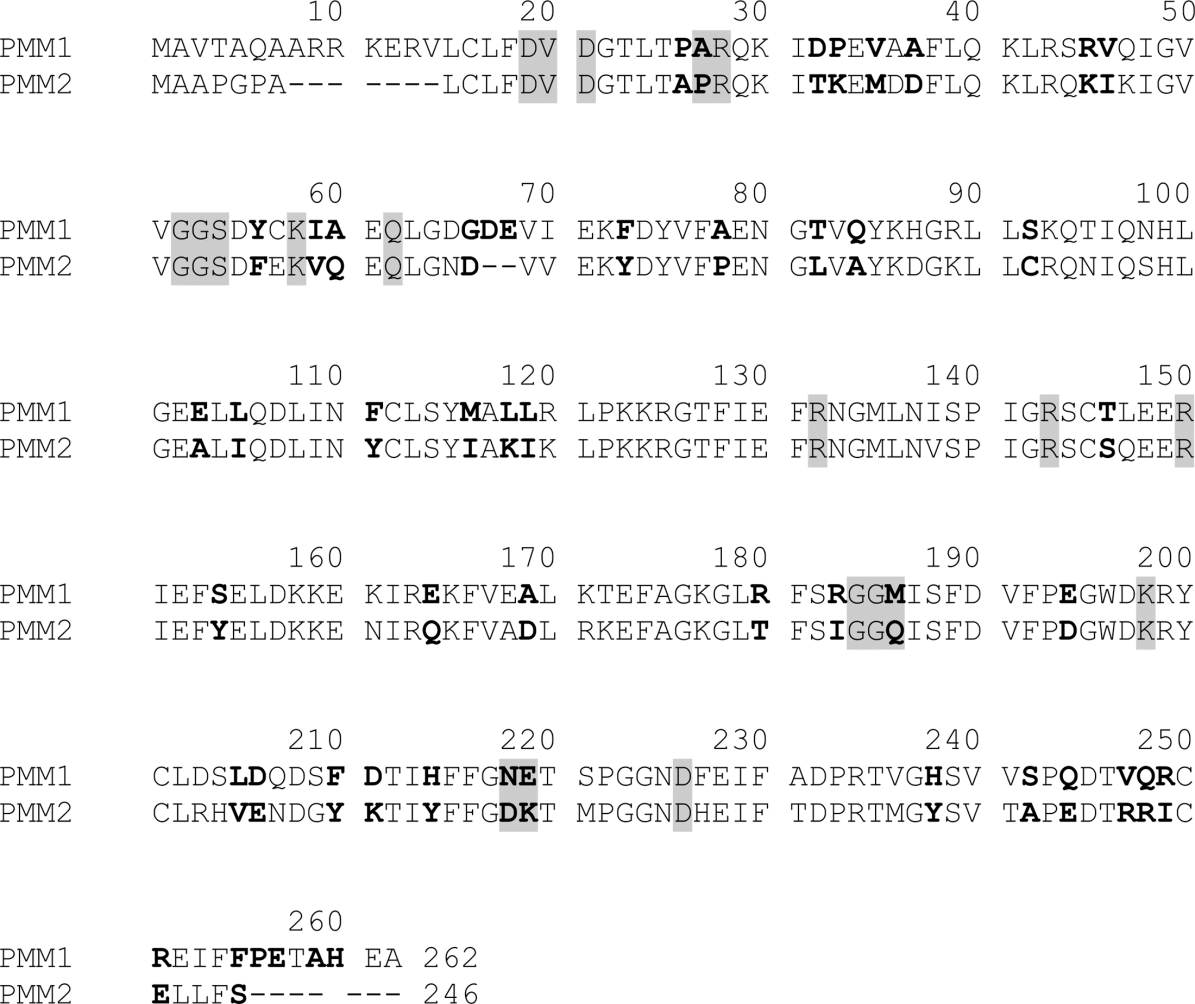
PMM1 and PMM2 sequence alignment. Active site residues are highlighted in grey, residues that are conserved in PMM1 orthologous proteins, but not in PMM2 orthologous proteins are in bold.

NMR experiments prove that both PMM1 and PMM2 convert Man-6-P into Man-1-P and confirm that only PMM1 has a relevant IMP dependent phosphatase activity. In order to clarify the molecular determinants of this difference, the sequences of PMM1 and PMM2 were aligned (Fig 4) and the residues that line the active site of PMM2, were highlighted in grey. The residues that are conserved in PMM1 and in its homologs, but not in PMM2s are in bold. Three residues belonging to the active site and not conserved in PMM1, could be responsible for the functional differences between human phosphomannomutases. The triple mutant M186Q-N218D-E219K-PMM1 (QDK-PMM1) was produced in order to test this hypothesis.

In Table 1 we compared the main functional parameters of PMM2, PMM1 and QDK-PMM1. In this case initial velocity are measured using standard coupled assays which can only monitor the conversion of Man-1-P into Man-6-P or that of Glc-1-P into Glc-6-P. We compared the phosphomannomutase activity of the enzymes under saturating conditions **(**200μM Man-1-P, 100μM Man-1,6-P_2_): PMM2 67.0 ± 3.1 U/mg, PMM1 17.0 ± 0.9 U/mg, QDK-PMM1 9.6 ± 0.4 U/mg.

**Table 1:** Functional parameters of PMM1, PMM2 and QDK-PMM1. Enzymatic activities were measured at the specified conditions and results were expressed as % of the PMM activity. *K*_M_ and *K*_a_ were determined from a Michaelis-Menten plot.

parameter	Experimental conditions	PMM2	PMM1	QDK-PMM1
PMM activity	200μM Man-1-Psaturating Man-1,6-P_2_	100 ± 9	100 ± 10	100 ± 8
PGM activity	600μM Glc-1-P saturating Glc-1,6-P_2_	5.8 ± 0.6	33.5 ± 3.5	41.7 ± 3.8
phosphatase activity	145μM Glc-1,6-P_2_	0.013 ± 0.002	2.35 ± 0.21	4.59 ± 0.45
phosphatase activity	80μM Man-1,6-P_2_	0.015 ± 0.002	2.03 ± 0.65	1.50 ± 0.31
phosphatase activity	145μM Glc-1,6-P_2_ 170μM IMP	0.014 ± 0.004	40.6 ± 15.9	4.20 ± 1.63
phosphatase activity	80μM Man-1,6-P_2_ 170μM IMP	0.016 ± 0.003	27.2 ± 5.0	1.52 ± 0.33
*K*_M_ Man-1-P, μM	10μM Man-1,6-P_2_ variable Man-1-P	23.7 ± 4.5 *	6.5 ± 1.3	1.6 ± 0.4
*K*_M_ Glc-1-P, μM	27μM Glc-1,6-P_2_ variable Glc-1-P	8.2 ± 0.7	7.3 ± 1.7	3.5 ± 1.3
*K*_a_ Man-1,6-P_2_, μM	200μM Man-1-P variable Man-1,6-P_2_	5.4 ± 0.6	17.0 ± 2.3	8.8 ± 1.2
*K*_a_ Glc-1,6-P_2_, μM	600μM Glc-1-P variable Glc-1,6-P_2_	9.0 ± 1.6	81.6 ± 12.4	23.2 ± 4.9
*K*_M_ Glc-1,6-P_2_, μM	variable Glc-1,6-P_2_	nd	3.9 ± 0.3	nd
*K*_a_ IMP, μM	150μM Glc-1,6-P_2_ variable IMP	nd	8.6 ± 2.3	nd

* 3μM Man-1,6-P_2_

nd, not determined

**Fig 5:**
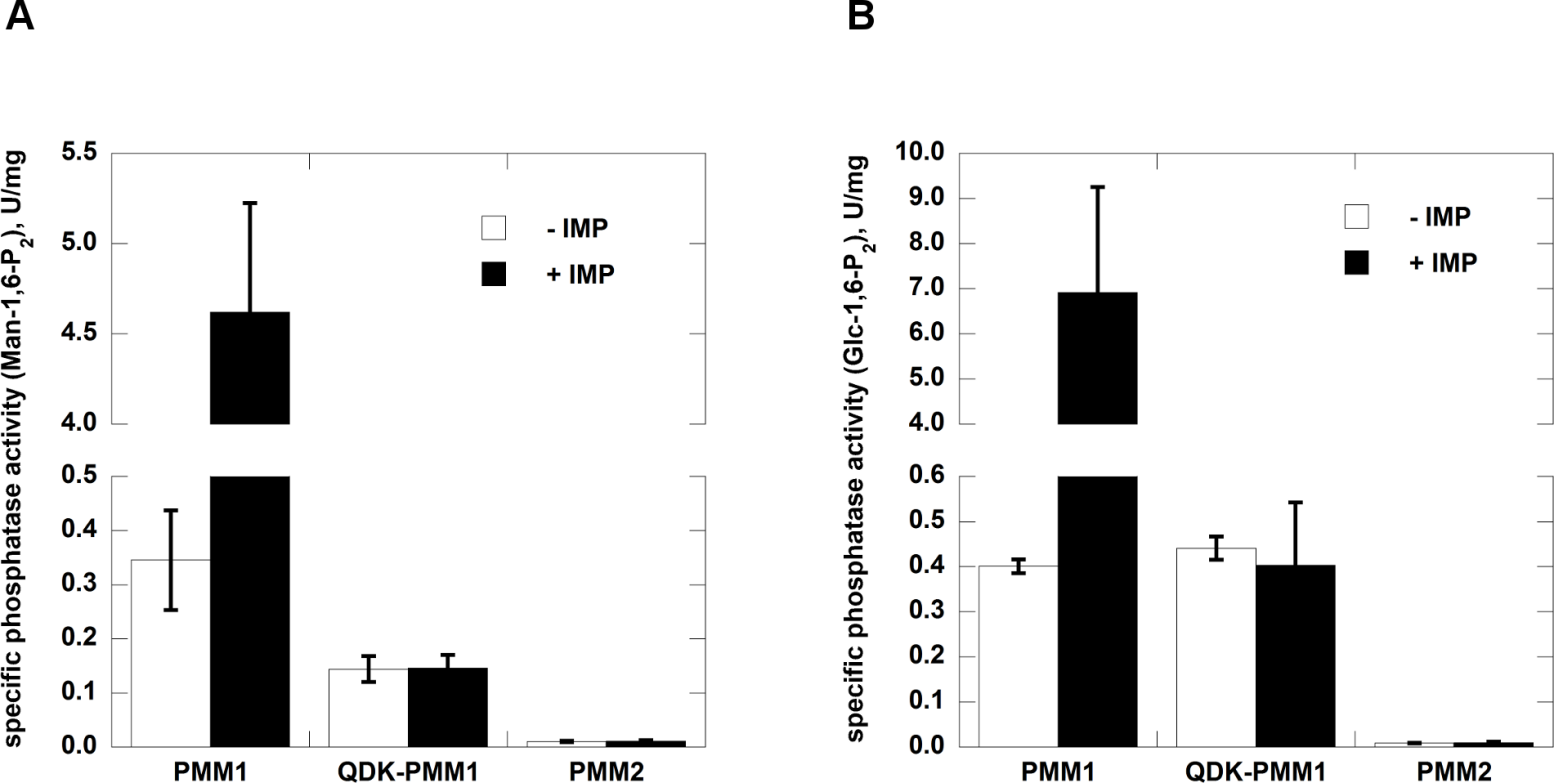
Effects of IMP on the phosphatase activity of PMM1, QDK-PMM1 and PMM2. Panel A) The substrate was Man-1,6-P_2_ 0.08 mM with or without IMP 0.17 mM. Panel B) The substrate was Glc-1,6-P_2_ 0.145 mM with or without IMP 0.17 mM.

The phosphommanomutase activity of PMM2 greatly exceeds phosphoglucomutase activity (approximately 20 times) and this preference is less pronounced with PMM1 and QDK-PMM1. PMM2 does not hydrolyze Glc-1,6-P_2_ or Man-1,6-P_2_ whereas PMM1 and QDK-PMM1 do. Up to this point QDK-PMM1 behaves like PMM1 although it shows higher affinity for substrates. The most striking difference between the mutant and its wild type counterpart is observed when the phosphatase activity is measured in the presence of IMP. QDK-PMM1 is insensitive to the activator (Table 1 and in Fig 5).

**Fig 6:**
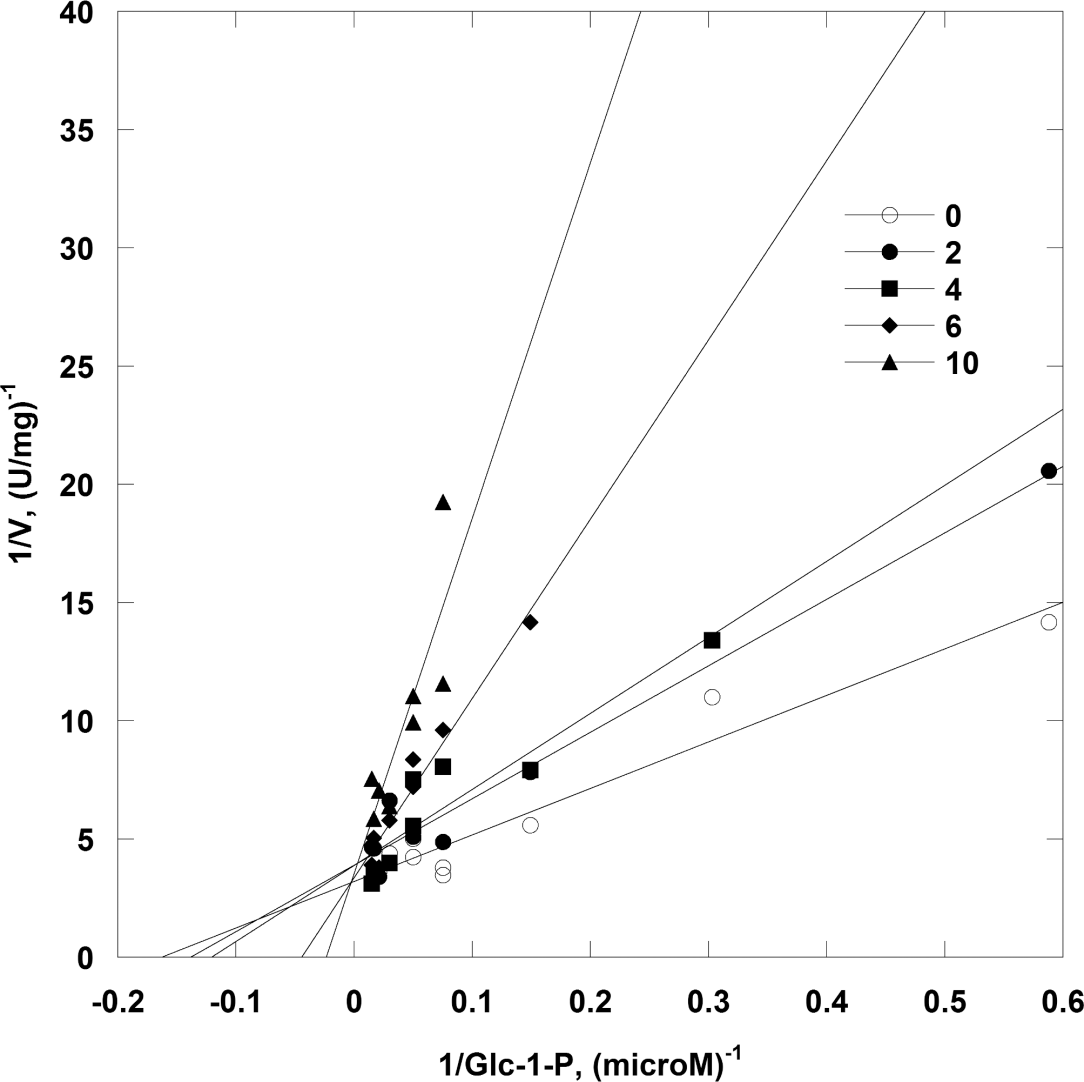
Competitive inhibition of PMM1 mutase activity by IMP. Phosphoglucomutase activity of PMM1 (Glc-1,6-P2 1 μM and Glc-1-P ranging from 0 to 60 μM) was measured at 0, 2, 4, 6 and 10 μM IMP. Data are shown as Lineweaver-Burk plots.

IMP acts as a switch because it enhances phosphatase activity and inhibits competitively the mutase activity of PMM1 (Fig 6).

**Fig 7:**
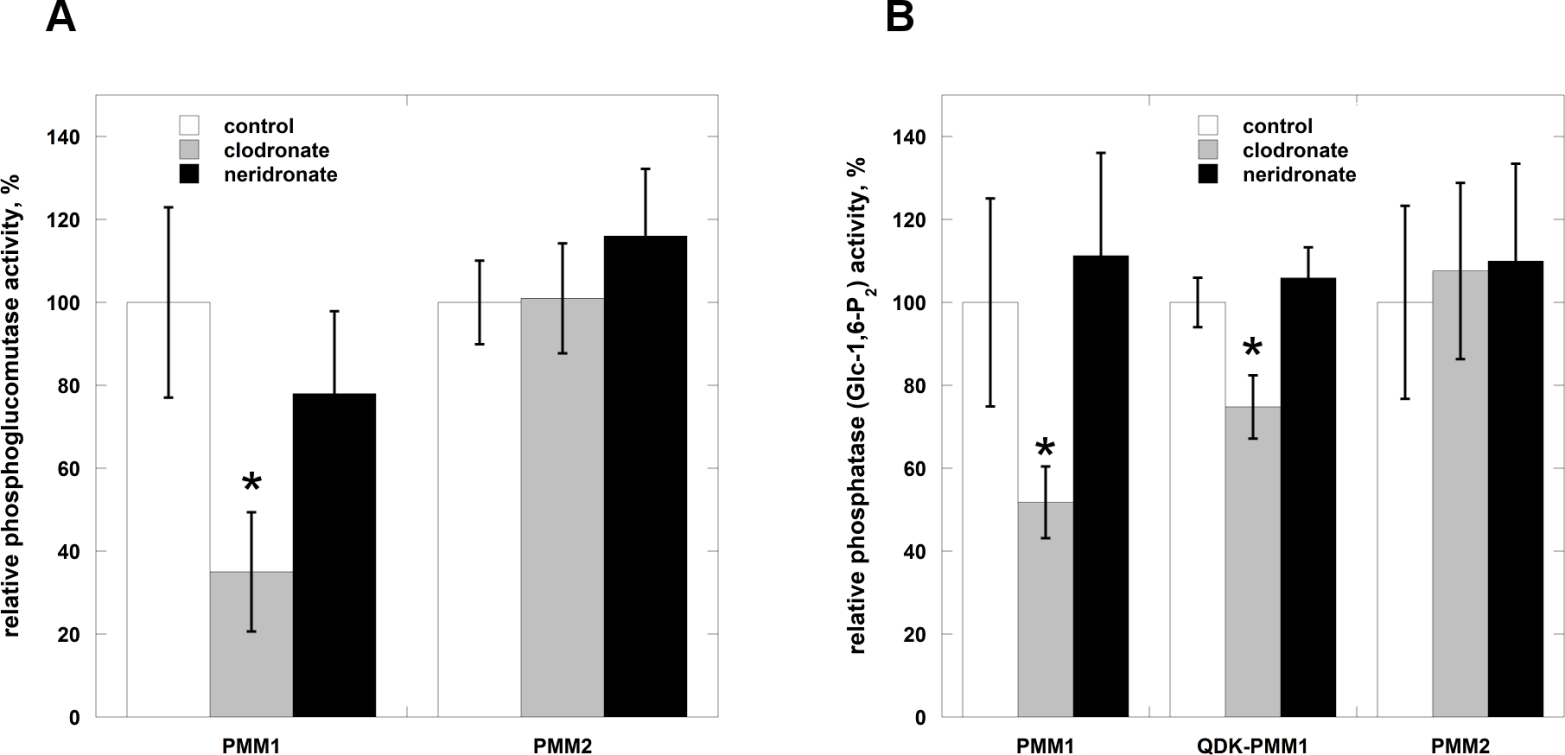
Effects of bisphosphonates on the phosphatase and glucomutase activities of PMM1, QDK-PMM1 and PMM2. Panel A) Phosphoglucomutase activity of PMM1 and PMM2 was measured in the presence of Glc-1-P 40 μM and Glc-1,6-P_2_ 27 μM. Panel B) Glc-1,6-P_2_-phosphatase activity of PMM1, QDK-PMM1 and PMM2 was measured in the presence of Glc-1,6-P_2_ 0.145 mM. In both cases the activities were also measured in the presence of clodronate (2.8 mM) or neridronate (1.5 mM).

The opposite effect on the two activities is peculiar and other inhibitors do not work in the same way. We looked among FDA approved molecules in order to use drug repositioning [35] and we identified the bisphosphonate clodronate as a novel specific inhibitor of PMM1 that decreases both PGM and Glc-1,6-P_2_-ase activities. Another bisphonate, neridronate does not inhibit either PMM1 or PMM2 (Fig 7).

**Fig 8:**
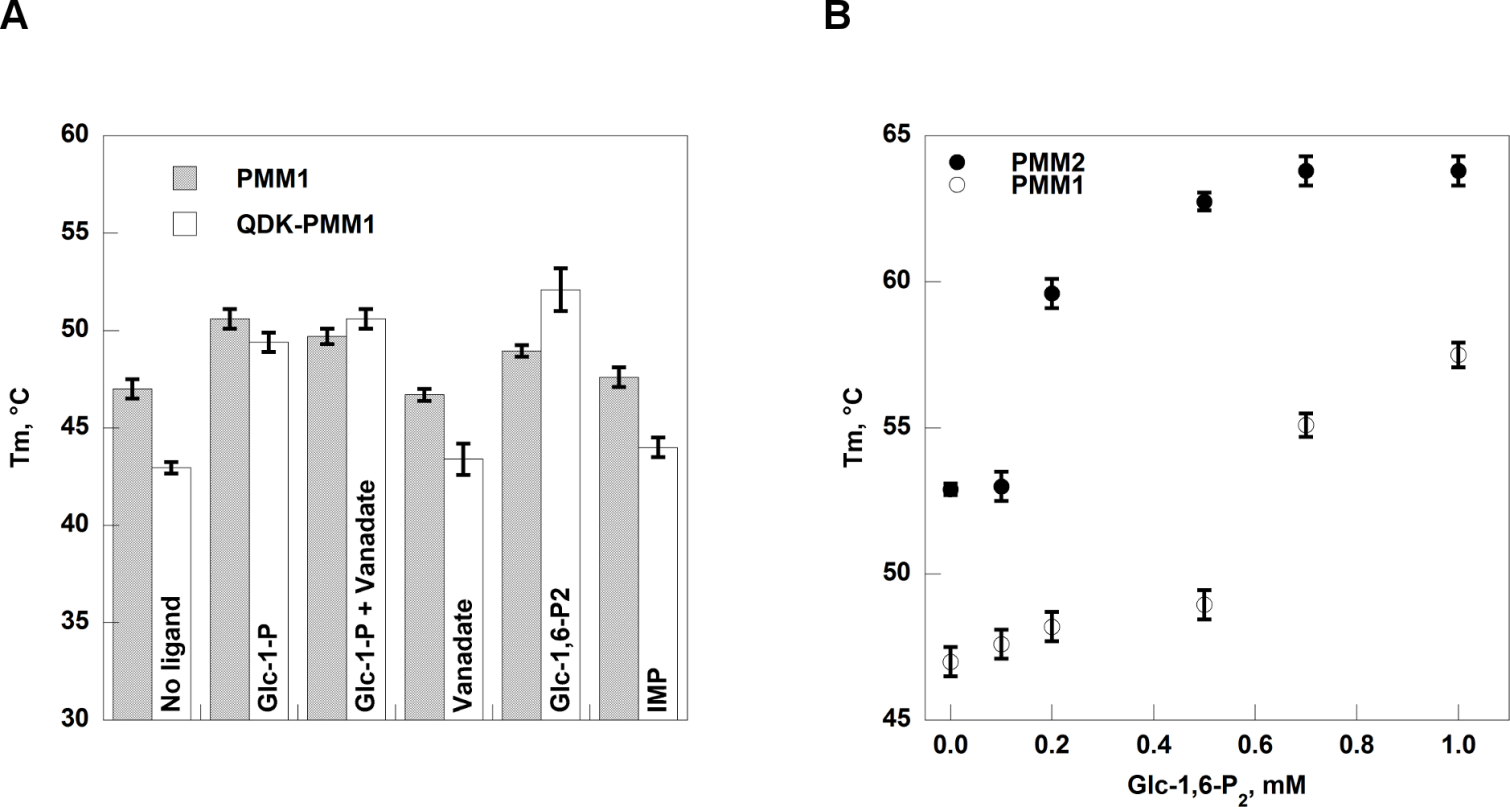
Thermal stability of phosphomannomutases. Panel A) Melting temperatures of PMM1 and QDK-PMM1 (0.5 mg/ml) were measured in the presence of different ligands: Glc-1-P 0.5 mM, Glc-1-P 0.5 mM + vanadate 0.5 mM, vanadate 0.5 mM, Glc-1,6-P_2_ 0.5 mM, IMP 0.17 mM. Panel B). Melting temperatures of PMM1 and PMM2 (0.5 mg/ml) were measured in the presence of different concentrations of Glc-1,6-P_2_ (ranging from 0 to 1 mM). The experiments were conducted at pH 7.5 in the presence of 2.4x SyproOrange by Thermal Shift Assay and the temperature increase was 1°/min from 20 to 90°C.

The mutant QDK-PMM1 is a stable protein with a melting temperature of 43 +/- 0.3 degrees only slightly lower than wild type (Fig 8, panel A). The stabilizing effect of ligand binding is statistically significant for the mutant and less pronounced with wild type PMM1 (Fig 8, panel A) in accordance with the affinities measured by enzymatic assays (Table 1). The effect of Glc-1,6-P_2_ on the melting temperature of PMM1 is seen only at very high concentrations of the ligand. For a comparison we monitored the melting temperature of PMM2 as a function of Glc-1,6-P_2_ concentration (Fig 8, panel B). The bis-phosphate hexose binds PMM2 as previously shown by *in silico* docking [17].

### Structural characterization by in silico docking

**Fig 9:**
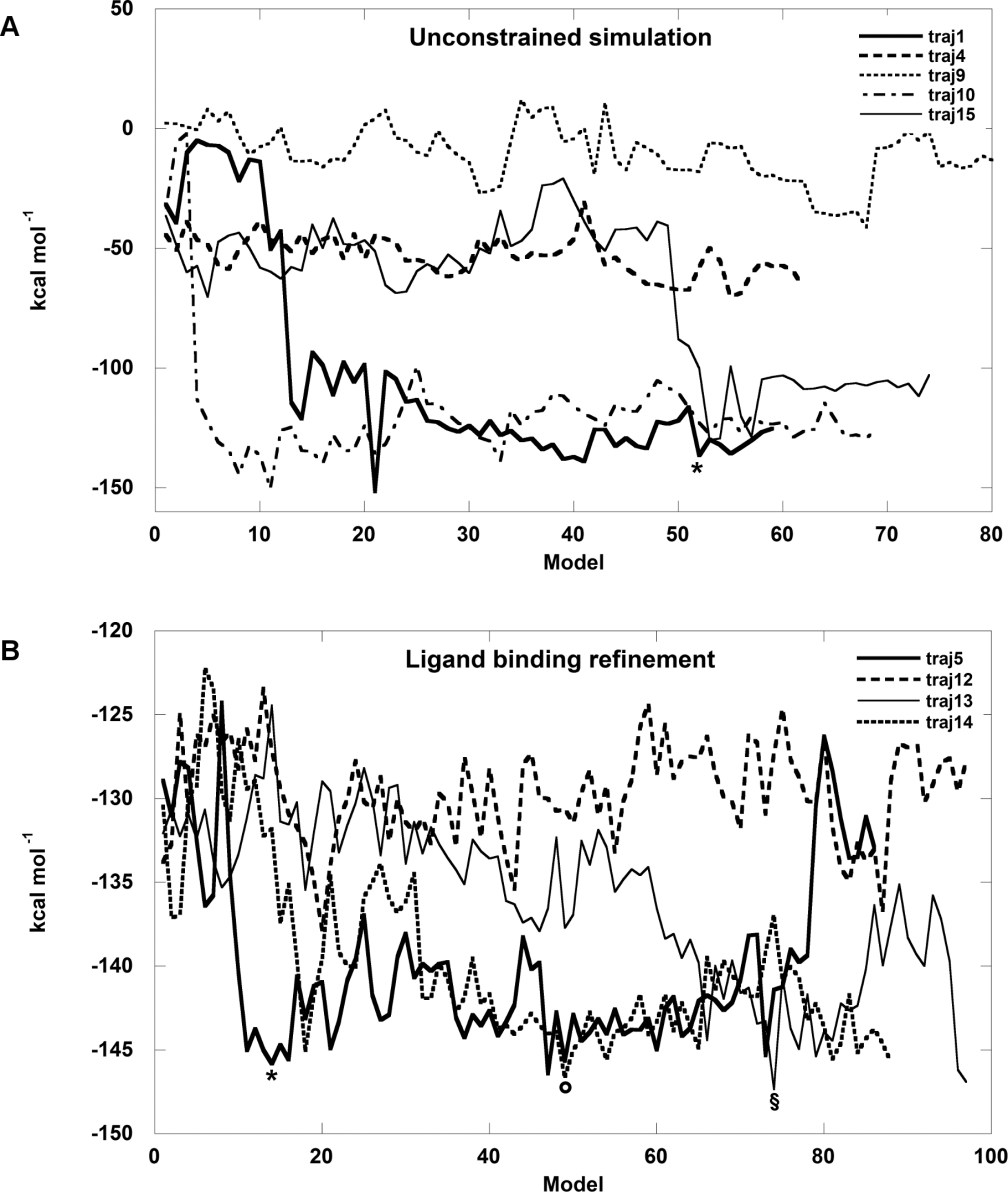
In silico docking of IMP and PMM1. Panel A) Unconstrained simulation. Only 5 trajectories are shown. Structure 52, used for the next refinement simulation, is identified by an asterisk. Panel B) Refinement simulation for structure 52 from traj1 in panel A. The circle, the section mark and the asterisk represent the structure further analyzed (see Fig 10 panel B).

**Fig 10:**
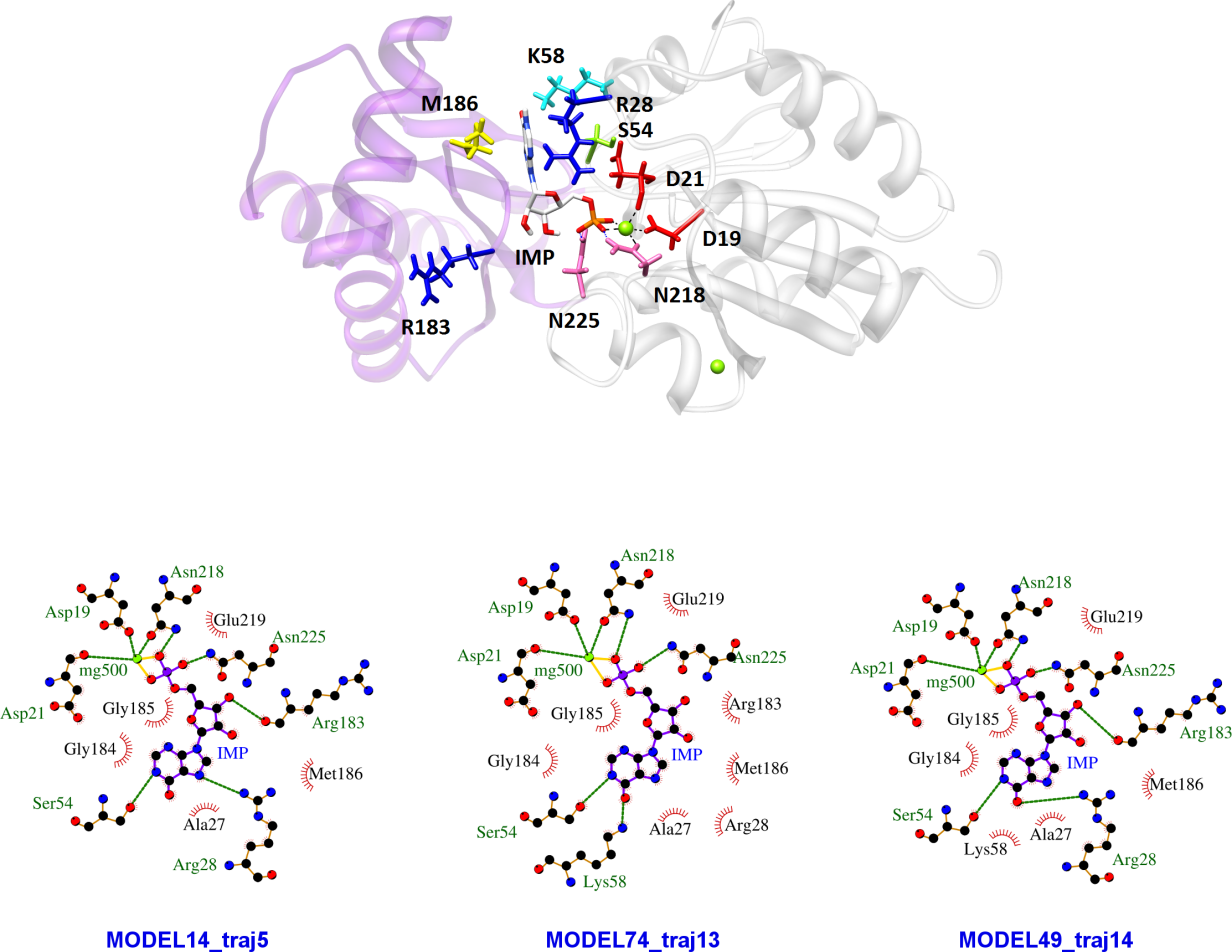
Ligand binding interactions. Panel A) A zoom-in of the interaction between IMP and PMM1 as it is seen in the low energy structure 52 (see Fig 7, panel A): the cap domain is shadowed in light magenta, the core domain is shadowed in grey. Panel B) Refined binding modes: details from energetic minima structures from 3 different trajectories (asterisk -traj5, section mark -traj13, circle -traj14 in Fig 9, panel B).

The biological assembly of PMM1 is a dimer where each subunit is made up of a cap domain (aa 95–194) and a core domain (aa 2–91, 199–262) connected by hinge peptides. It was obtained from the structure 2FUE and used as the input for IMP docking. In the first place an unconstrained simulation was run when the ligand is free to explore the protein surface. "PELE’s heuristic approach generates trial moves based on protein and ligand perturbations. The collection of accepted steps forms a stochastic trajectory" [29]. The ligand interaction energy was plotted for each step and for 15 trajectories, but in Fig 9 panel A only 5 trajectories are shown to exemplify the possible explorations of the ligand. When IMP does not find a binding site (traj9 in Fig 9, panel A), the energy fluctuates, but remains around -10 kcal/mol. When IMP reaches the cap domain, the energy reaches a plateau and fluctuates around the value of -53 kcal/mol (traj4 in Fig 9, panel A). The phosphate of IMP binds a patch of amino-acids, Ser188, Met186, Arg150, which lays opposite to the Mg^2+^ coordinated by Asp19, Asp21 and Asn218 where catalysis occurs. Silvaggi *et al*. [16] described the binding of Man-1-P at the same patch of amino-acids opposite to the Mg^2+^ catalytic center in the structure 2FUE and commented that the resulting complex could represent the first encounter of the enzyme and the ligand. When IMP reaches the core domain and the phosphate of IMP binds the Mg^2+^ catalytic center, the energy fluctuates around the value of -105 kcal/mol (traj10 in Fig 9, panel A). In some cases we observe the first encounter with the cap domain followed by the encounter with the core domain (traj15 in Fig 9, panel A). When the ligand bridges the core and cap domain the energy reaches the lowest plateau and fluctuates around -125 kcal/mol (traj1 in Fig 9, panel A). A zoom-in of the interaction between IMP and PMM1 as it is seen in the low energy structure (structure 52 in traj1 identified by an asterisk in Fig 9, panel A) is shown in Fig 10, panel A: the cap domain is shadowed in light magenta, the core domain is shadowed in grey. IMP binds the Mg^2+^ center and Asn218 with the phosphate and Met186 with the purine ring.

A refinement was run using structure 52 from traj1 as the input. The ligand interaction energy was plotted for each step and for 15 trajectories, but in Fig 9, panel B, only some examples are shown. In some cases, like in the case of traj 12 the energy fluctuates, but on average, does not decrease, in other cases, like in the case of traj5, traj13, traj14, the ligand binding energies decreases. We analyzed the structures from 3 different trajectories (traj5, traj13, traj14) corresponding to energetic minima (circle, section mark and asterisk in Fig 9, panel B). In the refined binding modes (Fig 10, panel B), which represent possible optimizations of the interactions observed after unconstrained docking (Fig 9, panel B), the Mg^2+^ catalytic center, Asn218 and Asn225 bind the phosphate. Ser54 and either Arg28 or Lys58 form hydrogen bonds with the purine ring. These residues are conserved in PMM1 and PMM2 (Fig 4). Arg183 and Met186, which are conserved in PMM1 family, but not in PMM2 family, are found in the proximity (>3.5 Å) of the IMP. Arg183 interacts by hydrogen bonds with the sugar using the backbone carbonylic oxygen.

## Discussion

The development of therapeutic interventions for PMM2-CDG is hindered by many basic questions still unanswered. One of these questions concerns PMM1, a paralog of PMM2. Using ^31^P-NMR we could monitor phosphomannomutase activity in the physiologically relevant direction for the first time and we demonstrated that PMM1 indeed produces Man-1-P and Man-1,6-P_2_ from Man-6-P and Glc-1,6-P_2_ as well as PMM2 does. Despite its ability to generate the metabolite needed for the synthesis of N- and C-glycosylated proteins and despite its expression in tissues that are affected in the disease, such as liver, lung, pancreas, and in particular in the brain [11, 36], PMM1 does not compensate for PMM2 deficiency in patients even if PMM2 activity is never completely absent. The residual activity of several mutants has been measured and compared to that of wt-PMM2 by Perez and co-workes [8] and by us, F119L 29% PMM activity, 42% PGM activity, V129M 89% PMM activity, 88% PGM activity, V231M 20% PMM activity, 20% PGM activity, (Andreotti unpublished).

The apparent paradox of two paralog enzymes, partly co-expressed, but not mutually compensatory, could be explained by differences in the affinities for substrates and relative PGM/PMM activities (Table 1 and [5, 6, 16, 37, 38]). However it is more likely that the ability to hydrolyse sugar bisphosphates represents the main physiological relevant difference between PMM1 and PMM2. Van Schaftingen and coworkers firstly demonstrated that PMM1, but not PMM2, has a phosphatase activity that is enhanced by IMP[7]. We showed that IMP is a reversible competitive inhibitor of PMM1 mutase activity. In accordance with this, *in silico* docking predicts that low energy binding of IMP occurs in the active site of PMM1. How is it possible that IMP inhibits one activity of PMM1 and promotes the other one? We noticed that contrary to what occurs with PMM2, which binds Glc-1,6-P_2_, closes up and is strongly stabilized against thermal denaturation [17], PMM1 is little stabilized by Glc-1,6-P_2_ and it is not stabilized by IMP. Our hypothesis is that PMM1 does not close as efficiently as PMM2 does and for this reason, the phosphorylated enzyme intermediate is more easily hydrolyzed. The binding of a bulky molecule such as IMP to the active site might further hinder the closure of cap and core domains. Inhibition of both activities occur with the small bisphosphonate clodronate.

If the main physiological function of PMM1 were its phosphatase activity, it could counteract PMM2 hydrolyzing Glc-1,6-P_2_. Hence it would be important to test whether reducing phosphatase activity, either inhibiting selectively PMM1 or acting on the IMP steady state concentration, is beneficial in cells with reduced PMM2 activity. In this paper we identified the residues that are responsible for the binding of IMP and demonstrated that it is possible to express a stable PMM1 mutant insensitive to the nucleotide. It could be possible to test the effect of IMP on N- and C- glycosylation, knocking out wt-PMM1 and expressing QKD-PMM1. On the other hand, the treatment with molecules that selectively inhibit PMM1, but not PMM2, such as clodronate, could help elucidate the effect of phosphatase activity on N-glycosylation.

## Abbreviations

PELE: protein energy landscape exploration
PMM: Phosphomammomutase
PGM: Phosphoglucomutase
Glc-1-P: Glucose 1-phosphate
Glc-1,6-P_2_: Glucose 1,6-bisphosphate
Glc-6-P: Glucose 6-phosphate
Man-1-P: Mannose 1-phosphate
Man-1,6-P_2_: Mannose 1,6-bisphosphate
Man-6-P: Mannose 6-phosphate
NADP^+^: β-Nicotinamide adenine dinucleotide phosphate
IMP: Inosine monophosphate
WT: wild type
